# Medication adherence among the elderly: applying grounded theory approach in a developing country

**DOI:** 10.1186/s40545-021-00340-9

**Published:** 2021-06-30

**Authors:** Peivand Bastani, Parisa Bikineh, Gholamhossein Mehralian, Omid Sadeghkhani, Rita Rezaee, Zahra Kavosi, Ramin Ravangard

**Affiliations:** 1 Health Human Resources Research Center, School of Management and Medical Information Sciences, Shiraz University of Medical Sciences, Shiraz, Iran; 2 Student Research Committee, School of Management and Medical Information Sciences, Shiraz University of Medical Sciences, Shiraz, Iran; 3 Pharmacoeconomics and Pharma Management Department, School of Pharmacy, Shahid Beheshti University of Medical Sciences, Tehran, Iran

**Keywords:** Medication adherence, The elderly, Grounded theory, Iran

## Abstract

**Background:**

Medication adherence is an important concept particularly among the elderly that can, directly and indirectly, affect the health system’s costs and the elderly’s health, quality of life, and functional abilities. This study aimed to determine the model of medication adherence among the Iranian elderly using the grounded theory approach.

**Methods:**

The concept of medication adherence and the determination of its process among the elderly is a multidisciplinary social issue that can be affected by many contextual factors. Grounded theory with the approach of Strauss and Corbin (2004) was applied to determine the customized model. Data triangulation occurred through semi-structured interviews, observation, field notes, and memoing. Open coding, selective coding, and axial coding were applied to analyze the data.

**Results:**

Delinquency in the medication use among the elderly was caused by factors such as doubtfulness, fear of complications, not following the patients by the physicians, and negative others and medical staff’s impacts. During the process of medication adherence, the patient’s lack of knowledge, lack of sufficient education, inappropriate and restricted lifestyle, difficult living conditions, and social pressures imposed on individuals could exacerbate and worsen the delinquency in medication adherence. It should not be neglected that some other factors such as lack of an effective supervision system, lack of supportive organizations, stakeholders’ market-based behaviors, consumption inconvenience, consumption stress, hopelessness, and misunderstanding could also aggravate the delinquency.

**Conclusions:**

Although the proposed theory and model were customized and context-based for the Iranian elderly, in general, making positive changes in the process of adherence to the medication use among the elderly requires scientific and basic management and planning of its factors. It should be noted that making these changes requires some interventions in and cooperation of all levels of the country's health system, from the Ministry of Health and Medical Education to the individual level of the elderly.

## Background

Today, with the help of progress and socio-economic development, different societies reach a higher average age and bring more life expectancy to their citizens [1]. This improvement in living conditions and increase in life expectancy has led to the emergence of the phenomenon of aging in communities [2]. According to the definition of the World Health Organization, aging is a period of life that starts from the end of 60 years and the beginning of 61 years [3].

Currently, the highest population growth rate in the world belongs to the elderly (population 60 years and older) and the world's elderly population is expected to reach about two billion by 2050, three times the elderly population in 2000 [4]. Although the phenomenon of aging was first experienced in developed countries, it has also become apparent in developing countries in recent years [5].

According to official censuses, Iran, such as many European countries, is aging [6]. Iran's elderly population is expected to increase from 8% in 2007 to 22% in 2046. This demographic crisis will create a tsunami phenomenon for the elderly in Iran [7].

According to epidemiological evidence in the field of aging, with increasing age, most chronic and degenerative diseases, including cardiovascular diseases, cancers, chronic obstructive pulmonary diseases, dementia, and other degenerative conditions are seen in people [8]. According to other evidence, aging has a direct and increasing effect on the number of visits to the doctor and pharmacy [1], and the elderly need to take more medications than young and middle-aged people [9], which may be because of physiological changes due to aging and, consequently, increased susceptibility to various diseases [10]. Therefore, it can be said that the elderly are the largest group of medication users [11].

However, the use of medication and adherence to medication as prescribed is a matter that is related to all cultural, social, and religious aspects of the people [12]. Medication adherence is defined as the degree to which patients take medications as prescribed by their doctors and includes two dimensions of timely medication use and medication use based on how it is prescribed by the physician [13]. Although the issue of medication adherence is important for all groups, it is more important in the elderly and there is a need to ensure that the patients adhere to the medications as prescribed so that they can receive maximum therapeutic benefits [14]. Poor medication adherence can lead to the development of complications and diseases, decreased functional abilities and quality of life, increased treatment costs, increased use of expensive specialized medical resources, unnecessary medication changes, and increased length of hospital stay [15].

The World Health Organization has divided the various factors affecting medication adherence into five groups, including socioeconomic factors, factors related to the health care team and service delivery system, factors related to the disease, factors related to treatment, and factors related to the patient. Although some of these factors are unchangeable, those that are relevant to the patient can be changed by educating patients and increasing their knowledge [16].

The results of different studies have shown some reasons for medication non-adherence, including forgetfulness, lack of motivation, high medication prices, inadequate health literacy, inadequate medication regimen, complex medication regimen [17], level of education, health problems, frequency of doses, dissatisfaction with counseling and explanation of medications [18], distrust of the physicians and their diagnoses, indifference to the use of medications, medication side effects, religious considerations, and misunderstanding the physician's instructions [15].

However, the studies conducted in Iran have indicated that medication use, especially in the elderly, lacks the correct pattern, and efforts to correct this pattern have not been very successful [19, 20]. On the other hand, the researchers in their searches found that there was no coherent theory in the country appropriate to the indigenous conditions of the process of medication use in the elderly, which had shown the living conditions of the elderly in a macro perspective and with a scientific theory and also no research had been carried out with this approach. Therefore, this study aimed to determine the model of medication adherence in the Iranian elderly using the grounded theory approach.

## Methods

This was a qualitative study conducted using the grounded theory approach. The aim of grounded theory is mainly to generate a theory to determine a framework for the participants’ behaviors towards and social interactions with a phenomenon [21]. In the present study, this approach was used to conceptualize the phenomenon of medication adherence among the elderly population as a complex and multidisciplinary concept that could be affected by socio-cultural context. The study was conducted from October 2019 to July 2020 in Shiraz, Iran.

### Sampling, patient recruitment, participants’ selection

In the current study, the study population included physicians, pharmacists, and nurses who had a rich experience of working with the elderly population and their medication use, and the people over 60 years of age who had the experience of at least 1 year of routine medication consumption and their family or caregivers who were able to share their experiences in this area. This comprehensive population was considered to achieve data triangulation in this qualitative research [22]. The participants were initially selected using the purposeful sampling method according to their knowledge and experience of medication adherence in the elderly and the tendency to participate, then the participant selection continued using the snowball sampling and theoretical sampling methods based on the categories, sub-themes, and themes emerged. Sampling was continued up to achieving the theoretical saturation. Two pharmacists, two physicians, three nurses, 16 old people, and two of their children were included as the study participants.

### The interview guide, data collection

Data were collected through deep and semi-structured interviews by two of the researchers (PB and OS), observation, field notes, and memoing for a better understanding of medication adherence among the elderly. The interviews were focused on the experiences of the medical staff approach to the elderly in how to use medications as well as the elderly people’s experiences with the medication use. Some main questions were used as the topic guide, and each interview was conducted individually according to the type of participant's answer and considering the main research questions. The interview guide was initially prepared by implementing three open interviews with an old person, a physician, and a pharmacist who were not selected as the main participants. Then, the interview guide was finalized by the research team, and the meaningfulness of the questions was checked by a pilot interview. The questions were designed to be started from the most general main questions to the more specific and detailed ones (Supplement A). The interview sessions were started with the open and warming question of “please tell me about your experiences of how to use medications” or “would you please talk about your last experience of forgetting medication consumption?”. Then, according to the participants’ answers and the information given, some related probing questions were asked. Both the interviewers had been trained to hold the interview sessions in an interactive manner, try to make a trustful atmosphere, and persuade the participants to increase their collaboration.

According to the simultaneous analysis of the data, the questions of the next interviews were determined regarding the extracted subcategories. The duration of each interview was between 45 and 75 min, depending on the circumstances of the interviewee and the agreement of the parties. The number of interviews varied from 1 to 2 times (mostly once), and the additional interviews were conducted by telephone and in some cases in person to resolve some of the ambiguities. With a prior agreement with the participants, all the interview sessions were held in their workplace for the physicians, pharmacists, and the nurses, and in a pleasant and convenient environment such as a park for the old people.

### Data analysis

All the interviews were recorded using a voice recorder after obtaining the participants’ voluntary permission. All the audios were listened carefully several times and transcribed word for word. The data analysis process started through open coding from the texts through constant comparison of the data with the research questions until the researchers were made sure that the new data was a repetition of the old one and the new open codes could not be developed. At this stage, the saturation level was achieved. Then, axial and selective coding was used to build the main and core concepts and their relationships. The Strauss and Corbin (2004) approach was used for data analysis and development of the theory [23].

**Fig. 1:**
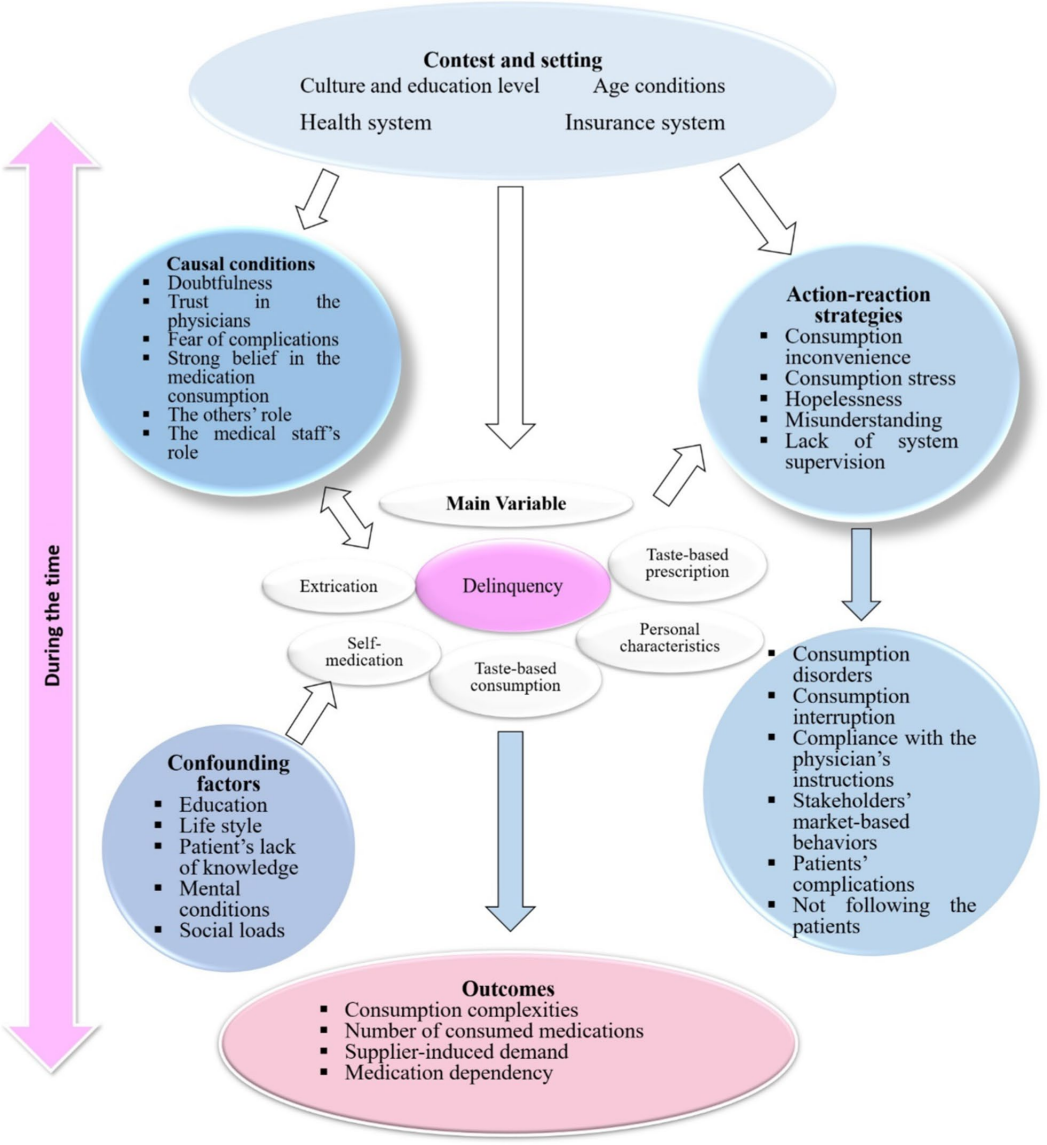
Outcome of the selective and axial coding according to the Strauss and Corbin diagram

- To do open coding, first, the text of the interviews and field notes were reviewed and re-read several times and the data were reviewed line by line and word for word, and the sentences and concepts in each line and paragraph were identified as the meaningful units that could to some extent answer the research questions, then, a code was assigned to each sentence or concept. An example table of the open coding process has been provided in Supplement B.
- Then, in the axial coding, the researchers seek to answer questions such as "why?", "How did it happen?", "Where?", "How?", "When?", and "With what results?". In response to these questions, the categories and their relationships were identified through continuous comparison of the emerged codes and their meanings and concepts. Through this process, categories with the higher level of conceptualization were made.
- Finally, using selective coding, the main category was identified and the relationship between the concepts and the initial framework of the theory was determined. Then, the researchers agreed on the core category and determined the relationship of other categories based on the suggested framework of Strauss and Corbin. The outcome of the selective and axial coding is presented in Fig. 1 according to the Strauss and Corbin diagram.

Four criteria of credibility, transferability, dependability, and conformability, proposed by Lincoln and Guba [24], were applied. In this regard, the long engagement of the research team with the research topic was led to the increase of credibility of the results. Data triangulation was also used to assure credibility. Audit trial and external check & debriefing were used to enhance the dependability. A deep description of the results was also helped to assure the transferability, and finally, for achieving the conformability, the objectiveness and neutralism occurred through the researchers and the experts’ agreement on the relations among the thematic model and the concepts. All the coding processes, from the initial open coding to the axial and selective coding for developing the framework, were performed by the authors (PB, RR) who do not have any conflict of interest to the subject. In addition, they had enough experience in the qualitative analyses for assuring the reflexivity of the study.

The study was approved by the Ethics Committee affiliated with Shiraz University of Medical Sciences (Code: IR.SUMS.REC.1398.541). The written informed consent was obtained from all participants.

## Results

The results of the present study were led to emerging 6 main themes and 23 sub-themes related to medication adherence among the Iranian elderly. These 6 main themes included “consumption disorders”, “delinquency”, “forgetfulness”, “consumption acceptance”, “socio-cultural factors” and “others’ impacts”.

According to the Strauss and Corbin (2004) approach, the context of the phenomenon, i.e., the medication adherence among the Iranian elderly, the causal conditions, action-reaction strategies, confounding factors, and the outcomes were emerged (Fig. 1).

According to Fig. 1, self-medication, taste-based consumption of medications, personal characteristics, taste-based prescription of the medications, and extrication were among the main issues emphasized by the participants that were considered as the concept of delinquency, as the main variable and challenge for medication adherence.

According to the results, different factors and organizations played an effective role in the process of medication adherence among the Iranian elderly that needed improvement, collaboration, and comprehensive planning. Moreover, better recognition of the context, including the community’s culture and education level, insurance system, health system, and age condition were among some of the noticeable issues. Meanwhile, it is important to know that some casual conditions such as the community’s doubtfulness, lack of trust in the health system, fear of complications, pressures from the others, some negative roles of the medical staff, and lack of belief in the medication consumption could worsen the delinquency in the medication adherence. Therefore, according to the results, better identification of the community’s capacity could help to apply action-reaction strategies and manage the confounding factors to decrease medication delinquency and increase medication adherence.

**Fig. 2:**
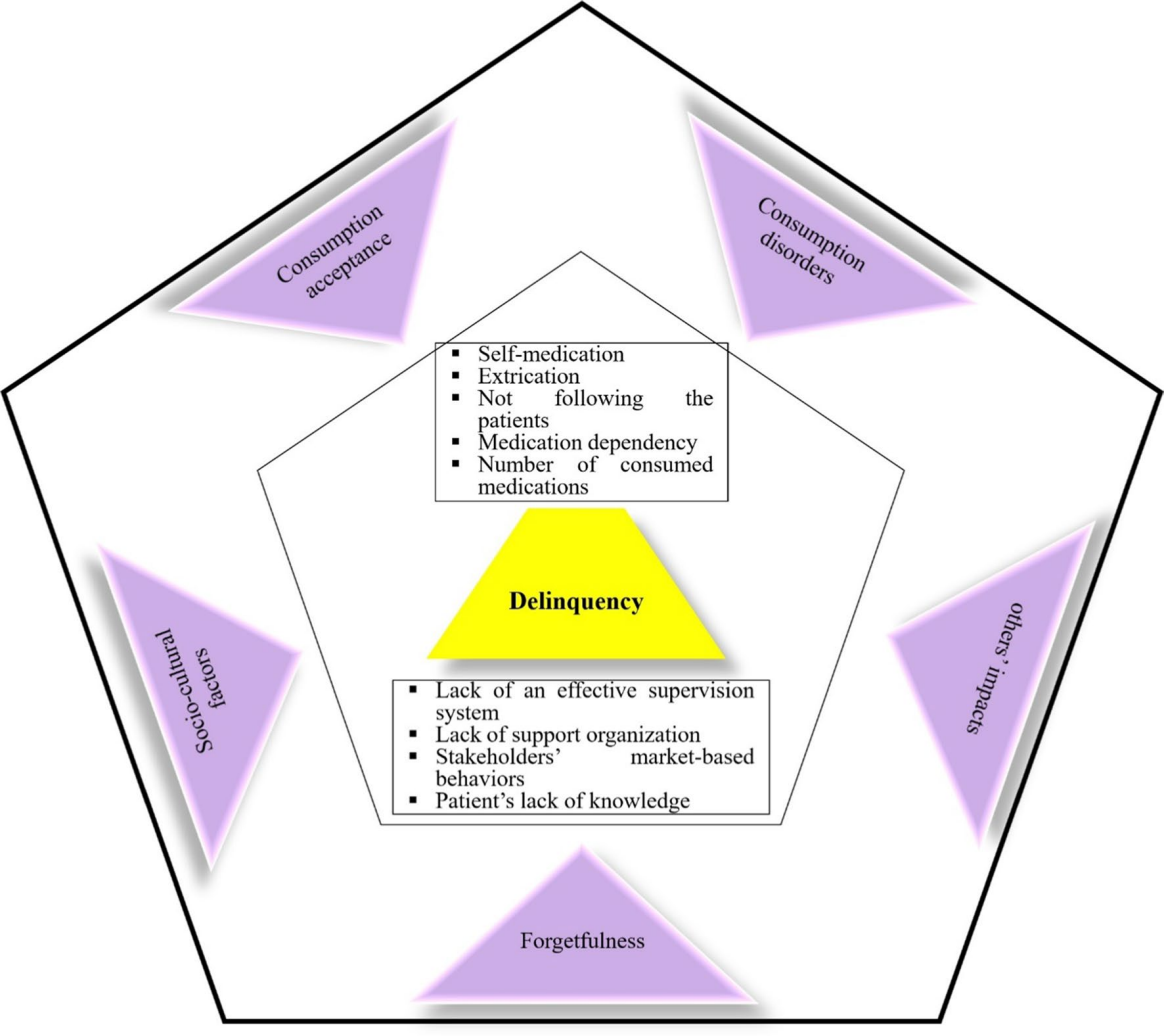
Delinquency theory of medication adherence among the Iranian elderly

The theory of delinquency is illustrated in Fig. 2. According to the results, delinquency in medication adherence among the elderly was caused by factors such as doubtfulness, fear of complications, not following the patients by the physicians, and negative others and medical staff’s impacts. During the process of medication adherence, the patient’s lack of knowledge, lack of sufficient education, inappropriate and restricted lifestyle, difficult living conditions, and social pressures imposed on individuals could exacerbate and worsen the delinquency in medication adherence. It should not be neglected that some other factors such as lack of an effective supervision system, lack of supportive organizations, stakeholders’ market-based behaviors, consumption inconvenience, consumption stress, hopelessness, and misunderstanding could also aggravate the delinquency.

## Discussion

Adherence to prescribed medications is defined as the degree to which a person's behavior conforms to medical and pharmacological advice. Adherence refers to a positive action in which the patient is sufficiently motivated to comply with the prescribed treatment because of a perceived advantage [25]. As the results of some studies in Iran, including Tavakoli’s study (2001), have shown that the medication use and adherence in the country, especially in the elderly, are not in a good situation, and the efforts made to correct them have not been very successful [19], the present study aimed to determine the model of medication adherence in the Iranian elderly to specify factors affecting the medication adherence of the elderly by considering the cultural, social, economic and local context in Iran using the grounded theory approach.

According to the results of the present study, delinquency was the main factor and problem in adhering to medications among the elderly. Other key themes that provided the model framework for adherence to medication use in the elderly included socio-cultural factors, consumption disorders, consumption acceptance, others’ impacts, and forgetfulness.

The results of the present study generally showed that many personal and external factors caused the elderly’s delinquency in the routine use of their medications, one of the most important of which could be the elderly’s taste-based consumption of medications as well as the physicians’ taste-based prescription of the medications for a particular disease and, most importantly, self-medication, which addressing these issues requires raising the level of awareness and knowledge of the elderly and trying to create more integration and coordination in the behavior of physicians. The results of studies by Jaam et al. (2017) [26], Murray et al. (2004) [27], and Salzman et al. (1995) [28] confirm the results of the present study.

According to the results of the current study, some other context-based factors affecting the medication adherence or delinquency among the elderly were the cultural background of the community, the existence of educational facilities, the level of health literacy in the community, the level of access to health care facilities, the type of health system, and the existing health insurance system, all of which were subthemes of socio-cultural factors, and strengthening each of them would improve the elderly’s adherence. Results of studies by Jaam et al. (2017) [26], O'quin et al. (2015) [29], Carpenter et al. (2014) [30], Galvan et al. (2014) [31], Holt et al. (2014) [32], Hatami et al. (2008) [33], Seyed Mirzaei (2007) [3], Delshad et al. (2005) [11], Murray et al. (2004) [27], and Zanjani (2001) [34] are in line with those of the present study. In this regard, it is suggested that considering the cultural and social capacity of the country, health policymakers should plan for education and empowerment of the elderly and also help to promote their level of self-sufficiency and self-care with a more comprehensive and practical perspective.

In addition, the results showed that consumption disorders could affect the medication adherence of the elderly. This factor can be due to both the innermost level of the medication adherence process, i.e., the individual level (including patient’s lack of knowledge and low level of health literacy), and the outermost level, i.e., the health system stewardship (including the cost of medications), and elimination of each of them requires comprehensive planning and cooperation of different organizations. Complications caused by the medication use (the intermediate level) are one of the factors affecting the behavior of the elderly in adhering to the medication use, which has been confirmed by the results of several studies [14, 35–39].

In addition, factors affecting consumption acceptance of the medications, such as fear of complications of not taking the medications and the insistence of others can affect the medication adherence of the elderly in such a way that the elderly are afraid of suffering from complications due to not taking the medications, or the experience of others about the side effects and complications of not taking medications will be a lesson for them. Moreover, the elderly may be encouraged to take medications because of the insistence of others and for receiving the attention and love of others. However, it should be borne in mind that excessive insistence or excessive commanding and prohibition by others can reduce the adherence to the medication use by the elderly. The results of the current study are similar to those of studies by Brown et al. (2016) [40] and Murray et al. (2003) [41]. In this case, justifying family members or caregivers and nurses of the elderly is of great importance that should be considered in the context of indigenous social and cultural capacities and constraints.

On the other hand, patients’ companions and medical staff, including physicians, can influence the elderly’s adherence to medication use. This result is consistent with the results of studies by Jaam et al. (2017) [26], Brown et al. (2016) [40], and Holt et al. (2014) [32]. In this regard, targeting empowerment courses for medical staff with a focus on the elderly and their medication needs can play an important role.

Finally, one of the most basic and important factors influencing the delinquency in the use of medications among the elderly was forgetfulness, which is natural and inevitable. In fact, decreased consciousness is an age-related issue that cannot be combated. However, to compensate for it, measures can be taken to reduce the elderly’s delinquency the medication use. Forgetfulness occurs for a variety of reasons, and identifying its causes and reasons is essential for providing the appropriate solutions. Because the interventions and solutions for a person who has forgotten to take medications due to the stress of life or work are different from those for a person who has a sensory disorder [40]. The results of the present study are in line with those of Holt et al. (2014) [32], Jolles et al. (2013) [42], and Salzman et al.’s (1995) [28] studies. To solve this problem, Zare et al. (2018) in their study have pointed out the need to take some measures to remind patients, including the use of brochures that can be installed on refrigerators and cabinets and sending reminder messages to patients about when to take medications [43].

In sum, the results of the current study tried to present a customized and context-based model for medication adherence among the Iranian elderly. According to this model, delinquency that was caused by the socio-cultural factors, consumption disorders, consumption acceptance, others’ impacts, and forgetfulness could highly be affected by the causal conditions and result in negative outcomes, such as supplier-induced demand, consumption complexities, number of consumed medications, and medication dependency. Such a model with all its factors and their relationships can pave the way for policymakers for better planning and designing and implementing more practical interventions.

To the best of our knowledge, there is no specific model developed for medication adherence among the elderly. Moreover, other general frameworks have not been achieved by the grounded theory and are not based on the contextual situation for developing countries. Finally, policymakers, providers, and managers in other developing, in transition, and under developed countries with similar cultural, social, economic, and technological contexts can benefit from applying the model provided in the present study in their real condition.

## Study limitations

This study, such as other studies, had some limitations, including being too busy and not having enough time to be interviewed by some participants, the need to interrupt and stop the interview when the clients and patients came due to conducting some interviews at the participants' workplace, and the need for a long-term presence of the researchers in the research environment to integrate the data obtained from observations and interviews.

## Conclusions

The results of the study showed that several factors affected the elderly’s medication adherence, among which delinquency was the main factor and problem. Considering the relationship between delinquency and other themes, and regarding the nature of customized and context-based theory and model presented in the present study, it seems that making positive changes in adherence to medication use among the elderly requires scientific and basic management and planning of these factors. It should be noted that making these changes requires some interventions in and cooperation of all levels of the country's health system, from the Ministry of Health and Medical Education to the individual level of the elderly.

## Data Availability

Are available from the corresponding author on reasonable request.
